# A potential marker of bare metal stent restenosis: monocyte count - to- HDL cholesterol ratio

**DOI:** 10.1186/s12872-016-0367-3

**Published:** 2016-10-03

**Authors:** Fatih Mehmet Ucar

**Affiliations:** Trakya University, Faculty of Medicine, Edirne, Turkey

**Keywords:** Novel marker, Monocyte /HDL cholesterol ratio, Instent restenosis, Bare metal

## Abstract

**Background:**

Oxidation and inflammation play significant roles in the pathogenesis of coronary artery diseases. Monocyte count to high-density lipoprotein (HDL) cholesterol ratio (MHR) is a new marker and has revealed as an indicator of inflammation in the literature. The present study aimed to search the effect of MHR on in-stent restenosis (ISR) in patients with stable or unstable angina pectoris undergoing bare-metal stent (BMS) implantation.

**Methods:**

A total of 468 consecutive stable or unstable angina pectoris patients (mean age 60.3 ± 10.1 and 70 % men) who had undergone successful BMS implantation were included the study. Serum samples were obtained before the procedure.

**Results:**

The mean period between two coronary angiography procedures was 14 ± 7.9 months. The baseline MHR levels were significantly higher in patients that had ISR (odds ratio, 3.64; 95 % confidence interval, 2.45- 4.84; *P* < 0.001). Stent diameter, the time between the two coronary angiographic studies, uric acid and MHR levels emerged as independent predictors of ISR.

**Conclusions:**

Our results indicate that elevated MHR is an independent and powerful predictor of ISR in patients with stable or unstable angina pectoris who underwent successful BMS implantation.

## Background

Despite new medications and techniques, in-stent restenosis (ISR) after successful percutaneous coronary interventions remains a major problem and limiting the efficacy of the procedure [1]. Inflammation, extracellular matrix remodeling, and smooth muscle cell proliferation are reasons for neo-intimal hyperplasia and restenosis [2, 3].

Circulating monocytes as a source of various cytokines and molecules, interact with platelets and endothelial cells and leading to aggravation of inflammatory, pro-thrombotic pathways [4]. High-density lipoprotein (HDL) defuse these pro-inflammatory and pro-oxidant effects of monocytes by inhibiting the migration of macrophages and oxidation of the low-density lipoprotein (LDL) molecules as well as promoting the efflux of cholesterol from these cells [5].

Monocyte count to HDL cholesterol ratio (MHR) has been reported as a new prognostic marker in cardiovascular diseases [6–8]. It has been revealed that MHR is related with major cardiovascular adverse events (MACE) including stent thrombosis and mortality after primary percutaneous coronary intervention (PCI) in ST-segment elevation myocardial infarction (STEMI) patients, [9]. To date, no study has evaluated the role of MHR in ISR. We hypothesized that pre-procedural MHR levels could predict ISR in stable or unstable angina pectoris patients who underwent bare-metal stent (BMS) implantation.

## Methods

### Study population

The study herein is a single-center and retrospectively analyzed data from 468 consecutive patients who underwent successful BMS implantation from January 2010 to December 2013 at our institution. The study was approved by the Ethics Commission of Turkiye Yuksek Ihtisas Training and Research Hospital and was implemented in complete concordance with the Declaration of Helsinki on human research. Patients who underwent PCI because of stable or unstable angina pectoris and successful BMS implantation were included in this study. Unstable angina pectoris were described according to the Braunwald classification [10].

Information including gender, age, smoking status, hyperlipidemia, hypertension, and diabetes mellitus was gathered. The definition of hypertension appeared as a systolic blood pressure value of ≥140 mmHg and/or a diastolic blood pressure value of ≥90 mmHg at least on >2 office BP measurements or being on an antihypertensive therapy. The definition of the diabetes mellitus comprised a blood sugar value of ≥126 mg/dl in the fasting state or being on an antidiabetic therapy whereas the status of hyperlipidemia was based on the presence of a total cholesterol level of ≥200 mg/dl or a triglyceride level of ≥150 mg/dl in the fasting state. Smoking was defined as current smoking or ex-smokers who forwent smoking in the past 6 months.

Severe liver disease, renal dysfunction (glomerular filtration rate <90 ml/min/1.73 m^2^) disease, active infections, heart failure, thyroid diseases, malignancy, autoimmune diseases or chronic connective tissue diseases, major surgery, trauma, and alcohol consumption were excluded from the study.

### Biochemical and hematological parameters

As part of our pre-procedural protocol, complete blood count, and biochemical parameters had already been available before coronary angiography for all patients. Dry tubes were used for biochemical analysis, and EDTA tubes were used for the hematological tests. White blood cell and erythrocyte counts, hemoglobin along with hematocrit levels were analyzed by an automated hematology device Coulter Counter LH Series (Beckman Coulter Inc, Hialeah, Florida). The biochemical measurements were determined by an automated analyzer of biochemistry (Abbott Aeroset, Abbott Laboratories, Abbott Park, Illinois).

### Coronary angiography and percutaneous coronary intervention

Coronary interventions were performed according to current practice guidelines and recorded in digital storage for further analysis [11]. The degree of coronary stenosis was visually estimated by experienced interventional cardiologists. Luminal narrowing > 50 % in a major sub-epicardial vessel (left anterior descending, left circumflex, or right coronary artery) was defined as significant stenosis. Target lesions were treated by elective PCI with coronary stent implantation. Only patients received the study which used BMS during the intervention and all BMS’s were thin-strut stainless steel stent. Each patient received 300 mg acetylsalicylic acid plus 300 or 600 mg clopidogrel before or during coronary intervention. Patients received weight-adjusted unfractionated heparin (100 U/kg) before the intervention. Femoral or radial access site for intervention was at the physician’s choice. Use of glycoprotein IIb/IIIa inhibitors and predilatation or postdilatation after stent implantation of the lesion was at the operator’s decision. A successful PCI was previously defined as the achievement of a minimum stenosis diameter reduction to <50 % in the presence of grade-III Thrombolysis in Myocardial Infarction flow (assessed by angiography) without side branch loss, flow-limiting dissection, or angiographic thrombus [12]. During routine clinical follow-up, coronary angiography was performed because of clinical indications in patients with stable or unstable angina pectoris. Control coronary angiograms were recorded using the Judkins technique and angiographic analysis was performed by two experienced and blinded interventional cardiologists. In the event of a disagreement, the assessment was then conducted by a third interventional cardiologist, and the final analysis was then considered. The ISR was defined evidence of stenosis ≥ 50 % either on the site of the previously treated vessel, inside the stent, or 5 mm proximally or distally to the previously treated vessel, requiring a new revascularization procedure of the target lesion [13].

### Statistical analysis

Continuous variables were expressed as mean ± standard deviation and categorical variables were defined as percentages (%). The chi-square test was used for the categorical variables. Data were tested for normal distribution using the Kolmogorov-Smirnov test. Continuous variables of normally disturbed variables were analyzed with an independent T-test, and continuous variables of non-normally disturbed variables were analyzed with Mann-Whitney U test. The Pearson correlation test was used for correlation analysis.

Predictors of ISR were determined by logistic regression analysis. Univariate linear regression analysis was used to evaluate the association of ISR with several biochemical and hematological parameters. All items with significant results in univariate analysis were included in multivariate analysis, followed by a stepwise forward elimination. The strength of association between variables and the ISR were represented by odds ratios and their accompanying 95 % confidence intervals. Receiver operating characteristic (ROC) curve analysis was used to determine the optimum cut-off level of pre-procedural MHR values to predict ISR. Statistical analyses were performed using SPSS 17.0 (SPSS Inc, Chicago, Illinois). A two-tailed p-value of <0.05 was regarded as statistically significant.

## Results

In the study population, mean age was 60.3 ± 10.1 years; clinical and procedural characteristics of the population according to with ISR and without ISR (*n* = 225 and 243, respectively) are summarized in Table 1. The lesion characteristics and angioplasty procedural variables were similar in both patient groups, except for stent diameter and time between the two coronary angiographic studies. Patients with ISR had narrower stent diameter compared with non-ISR group (2.8 ± 0.35 vs. 2.9 ± 0.35 respectively) (*p* = 0.01). The mean time interval from stent implantation to re-angiogram was 14 ± 7.9 months and the ISR group had significantly longer period between two coronary angiographies compared to non-ISR group (15.9 ± 8.5 vs. 13.1 ± 6.8 months, respectively <0.001 ).

**Table 1:** Basal demographic, clinical and angiographic features in coronary artery patients undergoing BMS implantation with and without ISR

	Non-ISR group (*n* = 243)	ISR group (*n* = 225)	P
Male, n (%)	158 (65)	171 (76)	0.15
Age (years, mean ± SD )	60.7 ± 10.0	60 ± 10.2	0.47
Hypertension, n (%)	152 (62)	122 (54)	0.06
Diabetes Mellitus, n (%)	73 (30)	69 (30)	0.88
Hyperlipidemia, n (%)	150 (61)	149 (66)	0.31
Smoke, n (%)	94 (38)	104 (44)	0.09
Left Ventricle Ejection fraction (%)	56.3 ± 4.5	56.0 ± 5.2	0.46
Target coronary artery, n (%)			0.43
-Left anterior descending	115 (47)	110 (49)	
-Left circumflex	76 (31)	50 (22)	
-Right	52 (22)	65 (29)	
Reason for stent implantation, n (%)			0.14
-Unstable angina pectoris	174 (72)	147 (65)	
-Stable angina pectoris	69 (28)	78 (35)	
Number of coronary arteries narrowed, n (%)			0.08
1	49 (20)	58 (26)	
2	165 (68)	148 (66)	
3	29 (12)	19 (8)	
Stent length (mm, mean ± SD)	15.8 ± 4.4	15.6 ± 4.1	0.60
Stent diameter (mm, mean ± SD)	2.9 ± 0.35	2.8 ± 0.35	**0.01**
Time between the 2 coronary angiographic studies (months, mean ± SD)	15.9 ± 8.5	13.1 ± 6.8	**<0,001**
Angiotensin-converting enzyme inhibitors, n (%)	195 (80)	192 (85)	0.14
Angiotensin receptor blockers, n (%)	15 (6)	10 (4)	0.40
Beta blockers, n (%)	202 (83)	195 (86)	0.28
Statins, n (%)	209 (86)	190 (84)	0.63

*ISR* in-stent restenosis, *BMS* bare-metal stenting, *mm* millimeter, *SD* standart deviation. Bold data displays statisticially significant difference (p<0.05)

**Fig. 1:**
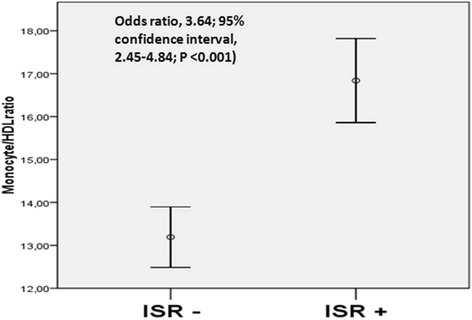
Median MHR values in patients with and without instent restenosis

The results of the hematological and biochemical parameters are listed in Table 2. Biochemical parameters were similar between groups except for HDL, C-reactive protein (CRP) and uric acid (UA) levels. Whereas the HDL levels were lower in ISR group than group non-ISR group (38.4 ± 9,3 vs. 41.9 ± 11.6) (*p* < 0.001) , the CRP (8.2 ± 8.79 vs. 5.8 ± 6.32) (*p* = 0.007) and UA levels (5.8 ± 1.48vs. 5.2 ± 1.45) (*p* < 0.001) were higher in ISR group compared to non-ISR group. The MHR was statistically higher in ISR group compared to non-ISR group (Fig. 1).

**Table 2:** Comparison of biochemical and hematological characteristics in CAD patients undergoing BMS implantation with and without ISR

	Non-ISR group (*n* = 243)	ISR group (*n* = 225)	P
Glucose, mg/dL, mean ± SD	120 ± 46.0	127 ± 52.5	0.11
Creatinine, mg/dL, mean ± SD	0.88 ± 0.26	0.91 ± 0.23	0.11
Total cholesterol, mg/dL, mean ± SD	177 ± 43.2	176 ± 43.1	0.77
Triglyceride, mg/dL	150 ± 77.5	164 ± 84.0	0.06
Low*-*density lipoprotein, mg/dL, mean ± SD	108 ± 34.9	106 ± 39.0	0.58
High-density lipoprotein, mg/dL, mean ± SD	41.9 ± 11,6	38.4 ± 9.3	<**0.001**
Uric Acid, mean ± SD	5.2 ± 1.45	5.8 ± 1.48	<**0.001**
Hemoglobin, g/dL, mean ± SD	13.9 ± 1.68	13.8 ± 3.21	0.76
Platelet, ×10^3^/L, mean ± SD	259 ± 74.6	267 ± 69.6	0.18
White blood cell, × 10 9 /μl , mean ± SD	7700 ± 1820	9100 ± 1730	<**0.001**
Neutrophil × 10 9 /μl , mean ± SD	4600 ± 1340	6600 ± 1770	<**0.001**
Lymphocyte ×10 9 /μl , mean ± SD	2200 ± 680	1700 ± 540	<**0.001**
Monocyte ×10 9 /μl , mean ± SD	550 ± 250	610 ± 240	**0.006**
C-reactive protein (mg/dl), mean ± SD	5.8 ± 6.32	8.2 ± 8.79	**0.007**
Monocyte/ HDL Ratio	14.1 ± 7.17	16.7 ± 7.95	<**0.001**

*CAD* coronary artery disease, *ISR* instent restenosis, *BMS* bare-metal stent, *HDL* high-density lipoprotein, *SD* standart deviation, *IQR* interquantile range. Bold data displays statisticially significant difference (p<0.05)

**Fig. 2:**
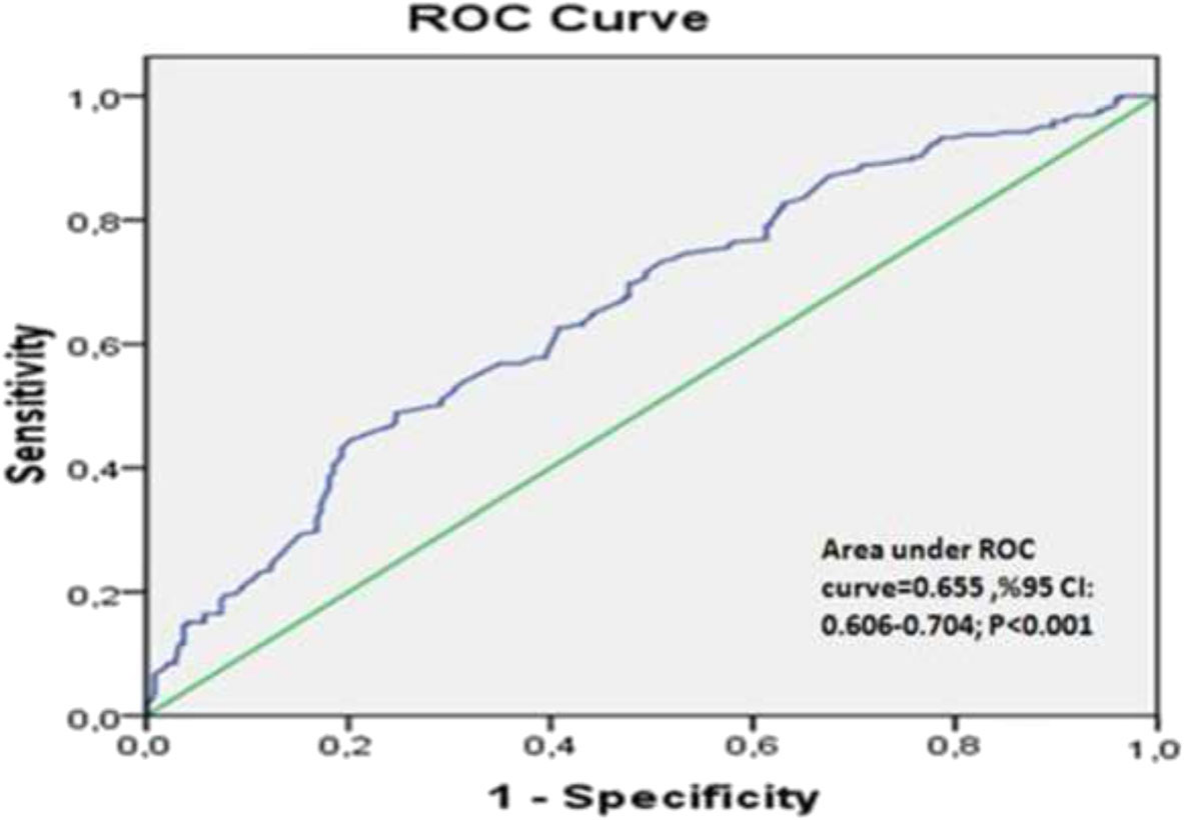
Receiver operating characteristic curve pre-procedural MHR and instent restenosis

Univariate logistic regression analyses showed that the stent diameter, the time between the two coronary angiographic studies, glucose, hemoglobin, CRP, serum UA and MHR levels were all significantly associated with ISR. These variables were included in a multivariate regression modeling, which revealed that stent diameter, the time between the two coronary angiographic studies, serum UA and MHR levels were independently associated with ISR (Table 3). Receiver operating characteristic curves were used to explore the relation between MHR and ISR. The area under the curve was 0.655 (95 % CI 0.606 to 0.704, *p* < 0.001). Using a cutoff level of 14.1, the pre-procedural MHR predicted ISR with a sensitivity of 66 % and specificity of 61 % (Fig. 2).

**Table 3:** Univariate and multivariate Cox regression analyses for predictors of bare metal stent restenosis

Variable	Univariate analysis			Multivariate analysis		
	HR	(95 % CI)	*p* value	HR	(95 % CI)	*p* value
Age	1.00	0.97–1.03	0.977			
Male gender	0.59	0.26–1.34	0.209			
Hypertension	1.20	0.66–2.17	0.538			
Diabetes mellitus	1.92	0.97–3.80	0.060			
Smoker	0.76	0.39–1.49	0.436			
Stent length	0.96	0.90–1.03	0.296			
Stent diameter	0.33	0.15–0.72	**0.005**	0.42	0.20–0.85	**0.017**
Time between 2 KAG	0.95	0.91–0.98	**0.004**	0.94	0.91–0.98	**0.002**
Glucose	1.00	1.00–1.01	**0.018**	1.00	099.–1.00	0.180
Creatinine	1.59	0.39–6.34	0.512			
HDL	0.97	0.95–1.00	0.155			
LDL	1.00	0.99–1.01	0.116			
Triglyceride	1.00	0.99–1.00	0.678			
LVEF	1.02	0.97–1.08	0.320			
Haemoglobin	0.82	0.67–1.00	**0.050**	0.92	0.78–1.08	0.327
MHR	1.05	1.00–1.11	**0.041**	1.09	1.04–1.14	**<0.001**
CRP	1.04	1.00–1.09	**0.024**	1.03	0.99.–1.07	0.051
Uric acid	1.23	1.01–1.50	**0.037**	1.24	1.04–1.48	**0.014**

*HDL* high density lipoprotein, *LDL* low density lipoprotein, *LVEF* left ventricle ejection fraction, *MHR* monocyte to HDL cholesterol Ratio, *CRP* C reactive protein, *HR* hazard ratio, *CI* confidence interval.Bold data displays statisticially significant difference (p<0.05)

## Discussion

The present study demonstrated that pre-procedural MHR levels are higher in patients with ISR compared to patients without ISR, and that MHR correlates with UA and CRP levels. Moreover, the MHR was an independent predictor of ISR on top of high CRP, UA levels, stent diameter and time between two coronary angiographic studies. The pre-procedural MHR >14.1 predicted ISR with a sensitivity of 66 % and specificity of 61 %. This might reportedly be the first study demonstrating the link between MHR and bare-metal restenosis in stable or unstable angina pectoris patients.

The patients with ISR had a higher risk of cardiac death and MI and that a prolonged regiment of DAPT may reduce this risk [14]. Despite new medications and techniques, however, ISR still remains an unresolved problem. After stent implantation, several processes play roles in the coronary wall [15]. During stent implantation, mechanical vascular damage occurs and triggers inflammation, followed by smooth muscle cell migration and proliferation. After that neo-intimal proliferation occurs and finally this process end with ISR by promoting neo-intimal proliferation through the stent struts [16, 17], particularly in patients with persistent systemic inflammation or autoimmune disease. Indeed, the inflammation is associated with many different diseases or disorders such as diabetes-associated nephropathy and retinopathy, infectious diseases, rheumatoid arthritis, systemic lupus erythematosus; chronic obstructive pulmonary disease (COPD); metabolic syndrome, end stage renal disease (ESRD) as well as various cardiovascular diseases or disorders including atherosclerosis. Moreover, it is well established that diabetes, ESRD or COPD are comorbidities related to higher ISR risk [18]. Therefore; several inflammatory markers have been studied in the prediction of ISR [19–21]. It has been demonstrated in a metaanalysis that pre-procedural CRP levels were a significant predictor of ISR after BMS implantation [22]. The count of circulating monocytes, as the source of tissue macrophages and foam cells, interact with platelets and endothelial cells and leading to aggravated inflammation [4]. Monocyte count has been shown as a predictor of coronary events [23] and monocyte activation is a very important step in the beginning of the atherosclerotic process [24]. HDL cholesterol has antiinflammatory, antioxidant, and antithrombotic effects [25]. HDL is highly effective at inhibiting endothelial expression of adhesion molecules and preventing monocyte recruitment to the artery wall [26]. HDL-cholesterol has a close interaction with monocytes through suppressing monocyte activities, interrupting differentiation of monocytes to macrophages which result in a restricted inflammatory response [27]. Therefore, it is possible to combine these two parameters into a single ratio as an MHR, which can reflect the underlying inflammation process. Indeed, it has been demonstrated that higher MHR related to worse cardiovascular events in chronic kidney disease [28] or the isolated coronary ectasia patients [29]. Canbolat et al. showed that pre-procedural MHR levels are associated with atrial fibrillation recurrence after ablation procedure [8]. In a recent study by Cetin et al., MHR was shown as an independent predictor of stent thrombosis in STEMI patients [9]. In all studies, the MHR was associated systemic inflammation and endothelial dysfunction and defined as a novel inflammation-based prognostic marker in cardiovascular diseases.

It is well known that another important role in the pathogenesis of ISR is oxidative stress. High levels of reactive oxygen species produced after PCI and they have been related to migration and proliferation of smooth muscle cells and resulted to ISR [30]. The UA is a well-known oxidative biomarker. Turak et al. demonstrated that the UA levels are related with ISR after BMS implantation and our findings are consistent with this [31]. Indeed, the oxidative stress and inflammation are closely related pathophysiological processes, one of which can be easily induced by another [32]. Since the role of inflammation as both a cause and a result of oxidative stress is supported by a considerable body of evidence, both processes are simultaneously found in many pathological conditions. On the other side, while the oxidative stress pathway is closely related to inflammation, most studies of biomarkers of oxidative stress do not consider markers of inflammation. Therefore, the combining this new MHR parameter as a “inflammatory marker” with the UA levels as an “oxidative stress marker” might be used in predicting of the ISR as a composite measure of oxidative stress- and inflammation-related restenosis process.

## Limitations

There are some limitations of the present study. Enrolment was retrospective and single-center design. The definition of ISR was based on visual assessment rather than more quantitative and informative intravascular ultrasound or optical computed tomographic results. Specificity and sensitivity of MHR in detecting ISR was relatively low. Also, other markers of oxidative stress and inflammation were not evaluated. This study only included patients with BMS stents, the findings should not be generalized to drug eluting stents. Notwithstanding these limitations, this is reportedly the first study focusing on MHR with regard to its predictive value in ISR after BMS implantation, and more comprehensive studies are still warranted to corroborate our findings.

## Conclusion

This is the first study to investigate the correlation between pre-interventional MHR and ISR after BMS implantation in patients with stable or unstable angina pectoris. Our results indicate that elevated MHR is associated with ISR, and showed a correlation between MHR and markers of inflammation (CRP) and oxidative stress (uric acid). Because of its wide availability and inexpensive cost for screening, pre-procedural MHR levels may identify patients at higher risk for ISR and may influence our clinical management.

## Abbreviations

BMS: Bare-metal stent
CRP: *C*-reactive protein
DM: Diabetes mellitus
HDL: High-density lipoprotein
HT: Hypertension
ISR: In-stent restenosis
MHR: Monocyte count to high-density lipoprotein cholesterol ratio
PCI: Percutaneous coronary intervention
STEMI: ST-segment elevation myocardial infarction
TIMI: Thrombolysis In Myocardial Infarction
UA: Uric acid
